# Asthma in 9-year-old children of subfertile couples is not associated with in vitro fertilization procedures

**DOI:** 10.1007/s00431-019-03436-2

**Published:** 2019-08-06

**Authors:** D. B. Kuiper, G. H. Koppelman, S. la Bastide-van Gemert, J. Seggers, M. L. Haadsma, T. J. Roseboom, A. Hoek, M. J. Heineman, Mijna Hadders-Algra

**Affiliations:** 1 University of Groningen, University Medical Center Groningen, Department of Paediatrics, Division of Developmental Neurology, Hanzeplein 1, Groningen, GZ 9713 The Netherlands; 2 University of Groningen, University Medical Center Groningen, Beatrix Children s Hospital, Department of Paediatric Pulmonology and Paediatric Allergology, Hanzeplein 1, Groningen, GZ 9713 The Netherlands; 3 University of Groningen, University Medical Center Groningen, Groningen Research Institute for Asthma and COPD, Hanzeplein 1, Groningen, GZ 9713 The Netherlands; 4 University of Groningen, University Medical Center Groningen, Department of Epidemiology, Hanzeplein 1, Groningen, GZ 9713 The Netherlands; 5 University of Groningen, University Medical Center Groningen, Department of Genetics, Division of Clinical Genetics, Hanzeplein 1, Groningen, GZ 9713 The Netherlands; 6 University of Amsterdam, Amsterdam University Medical Center, Department of Obstetrics and Gynaecology, Meibergdreef 9, Amsterdam, AZ 1105 The Netherlands; 7 University of Amsterdam, Amsterdam University Medical Center, Department of Clinical Epidemiology, Biostatistics and Bioinformatics, Meibergdreef 9, Amsterdam, AZ 1105 The Netherlands; 8 University of Groningen, University Medical Center Groningen, Department of Obstetrics and Gynaecology, Hanzeplein 1, Groningen, GZ 9713 The Netherlands

**Keywords:** Assisted reproductive techniques, Asthma, Asthma medication, In vitro fertilization, Ovarian hyperstimulation

## Abstract

Asthma is a chronic reversible obstructive airway disease, which is common among children and leads to respiratory impairment. Studies showed that asthma is more common among children born after in vitro fertilization (IVF) than among spontaneously conceived children. However, it is unknown which component of the IVF procedure contributes to this putative link. Therefore, the aim of this prospective follow-up study was to differentiate the possible effect of ovarian hyperstimulation from that of the in vitro culture procedure on asthma and rhinitis in 9-year-old children conceived with IVF. The study comprised three groups of singletons: (I) conceived with ovarian hyperstimulation-IVF (COH-IVF, *n* = 95); (II) conceived with modified natural cycle-IVF (MNC-IVF, *n* = 48); and (III) naturally conceived to subfertile couples (Sub-NC, *n* = 68). Parents filled out the validated Dutch version of the asthma questionnaire of the International Study of Asthma and Allergies. Asthma prevalence in the groups did not differ: COH-IVF *n* = 8 (8%); MNC-IVF *n* = 0 (0%); and Sub-NC *n* = 4 (6%). Adjustment for confounders did not alter the results.

*Conclusion*: Neither ovarian hyperstimulation nor the in vitro culture procedure was associated with asthma and rhinitis at 9 years. IVF children had a similar prevalence of asthma compared with children conceived naturally by subfertile couples.

*Trial registration*: ISRCTN76355836

**Table:** 

**What is Known:** • *An increased risk for asthma has been observed in children born after in vitro fertilization at preschool and school age.* • *The association between IVF and asthma may be partly explained by parental subfertility.*	
**What is New:** • *IVF children do not have a higher prevalence of asthma than children of subfertile couples conceived naturally.* • *Ovarian hyperstimulation used in IVF is not associated with asthma in 9-year-old children of subfertile couples.*

**Electronic supplementary material:**

The online version of this article (10.1007/s00431-019-03436-2) contains supplementary material, which is available to authorized users.

## Introduction

Worldwide, over 8 million children are conceived after in vitro fertilization (IVF) [4]. The short-term effects of IVF on children’s health have been studied comprehensively [13]. Overall, studies were reassuring; nevertheless, singletons conceived after IVF were more often born preterm and had a lower birthweight compared with natural conceived singletons [6]. Recently, studies increasingly address the possible effects of IVF on child health in later life [6]. One of the main points of interest of these studies is to clarify whether there is an association between IVF and atopic disease, including asthma and rhinitis [6]. Asthma is a chronic reversible obstructive airway disease, which is common among children and leads to coughing, wheezing, and breathing difficulties.

Recent studies found an increased risk for asthma in IVF offspring, independent from gestational age and birthweight [2, 5, 8, 9]. However, it is not clear yet which element of the IVF procedure (the in vitro culture procedure and/or ovarian hyperstimulation) may contribute to this putative link. Ovarian hyperstimulation induces the growth of multiple follicles, bypassing the natural selection of the development of one dominant follicle, and leads to higher oestrogen levels preceding the laboratory IVF procedures. During the in vitro culture procedure oocytes, sperm and embryos are handled outside the human body, possibly affecting their developmental phenotype. As it is known that the early environment of an embryo affects its physiology and thereby may lead to a higher risk for diseases in later life, it is conceivable ovarian stimulation and the in vitro culture procedure affect the health of the offspring [15]. Others suggested a link between parental subfertility and asthma in IVF offspring [7].

Therefore, the aim of this study was to disentangle the effect of ovarian hyperstimulation from that of the in vitro culture procedure independently from the effect subfertility on asthma and rhinitis in 9-year-old children. To this end, we compared three groups: (i) singletons conceived with controlled ovarian hyperstimulation-IVF (COH-IVF); (ii) singletons conceived with modified natural cycle-IVF (MNC-IVF); and (iii) naturally conceived singletons born to subfertile couples (Sub-NC). COH-IVF is the conventional form of IVF in which ovarian hyperstimulation is used. In MNC-IVF, the one follicle that naturally developed to dominance is used: no ovarian hyperstimulation is performed. The Sub-NC group comprised of children of all couples who achieved a singleton pregnancy while on the waiting list for fertility evaluation or treatment during the study period and wanted to participate in the study. These couples had been trying to conceive for at least 1 year; therefore, we expected parental characteristics to resemble the characteristics of IVF couples.

Differences between the COH-IVF and MNC-IVF group can be attributed to the effect of ovarian hyperstimulation. Comparing the MNC-IVF and Sub-NC group reveals the effect of the in vitro culture procedure.

## Methods

### Study design

The children participated in two parallel prospective studies: the Groningen assisted reproductive techniques (ART) cohort study and the preimplantation genetic screening (PGS) trial [10, 11].

The Groningen ART cohort study is a prospective assessor-blinded longitudinal follow-up study, which focuses on developmental outcomes and health of IVF offspring. The cohort started with subfertile couples who consulted the Department of Reproductive Medicine of the University Medical Center Groningen (UMCG). Couples with an expected delivery date between March 2005 and December 2006 were invited during the third trimester of pregnancy to participate in the study. The singletons formed the following three groups: (i) COH-IVF, (ii) MNC-IVF, and (iii) Sub-NC [11].

The PGS trial is a multicenter randomized controlled trial (Amsterdam University Medical Center (AUMC) and UMCG) comparing IVF with and without PGS. Women who received help conceiving by means of IVF at the Departments of Reproductive Medicine of the AUMC or UMCG between May 2003 and November 2005 were recruited [10]. Of the PGS trial, only those singletons who were conceived after COH-IVF without PGS (i.e. the control group) were eligible to participate in the current study. Twins, children born after oocyte cryopreservation and oocyte or embryo donation, were excluded. Information on the prenatal, neonatal, and perinatal period was collected on standardized charts 2 weeks after the expected delivery date. This included parental asthma, maternal BMI, smoking during pregnancy, and smoking in the household. The effect of the duration of subfertility was evaluated with the help of the proxy variable time to pregnancy (TTP). Information about TTP was retrieved from the medical file of the couples. In case of miscarriage TTP can be < 1 year, as TTP has a new onset [11].

At the 9-year follow-up assessment, parents filled out the validated Dutch adaption of the questionnaire used in the International Study of Asthma and Allergies (ISAAC), which was previously used in the Dutch national birth cohort Prevention and Incidence of Asthma and Mite Allergy (PIAMA) [1, 16]. The medical ethics committee of the UMCG approved the study design of the 9-year follow-up of the Groningen ART cohort study and PGS trial (reference number M09.074824; current controlled trial number, ISRCTN76355836) and parents provided written informed consent.

### Outcome measures asthma and rhinitis

The ISAAC questionnaire consists of questions about asthma, eczema, and rhinitis. Consistent with the asthma definition as defined by international experts in the Mechanisms of the Development of Allergy (MEDALL) study, asthma was defined as a positive response to at least two out of the three questions on asthma: (I) Did your child ever have asthma?; (II) Did your child have wheezing complaints in the past 12 months?; and (III) Did your child use asthma medication in the past 12 months? Rhinitis was defined as a positive answer to two specific questions in the questionnaire: (I) In the past 12 months, has your child had a problem with sneezing, or a running, or a blocked nose when he/she DID NOT have a cold or the flu? and (II) In the past 12 months, has this nose problem been accompanied by itchy-watery eyes? (for detailed information, see Asher et al. 1995 and Pinart et al. 2014) [1, 12].

### Statistical analysis

The original power calculation of the Groningen ART cohort study was based on neurological outcome at the age of 18 months. Our study was initially not designed to investigate the prevalence of asthma and rhinitis.

To estimate differences in background and outcome characteristics, univariable and multivariable statistics were used. The multivariable regression analyses adjusted for the following confounders: at least one parent with asthma, a currently smoking parent, birthweight, maternal body mass index, and TTP. The confounders were selected on a priori bases and in keeping with the literature [6, 13]. Results are expressed as odds ratio (OR) with 95% confidence interval (95% CI). The analyses were performed in SPSS Statistics version 20.0 (IBM Corp, NY, USA).

## Results

### Participation and parental and infant characteristics

Of the Groningen ART cohort study, 58 COH-IVF, 48 MNC-IVF, and 68 Sub-NC children participated. Of the PGS trial, 37 COH-IVF singletons took part in the study. This means that we assessed in total: COH-IVF *n* = 95, MNC-IVF *n* = 48, and Sub-NC *n* = 68 children (see supporting information flow chart). Overall, postnatal attrition was 22% and was nonselective. We did not find differences between the COH-IVF group of the Groningen ART cohort and the PGS trial regarding background and outcome characteristics.

The characteristics of participating parents and children of the three study groups are displayed in Table 1. Parents of COH-IVF singletons were older than those in the MNC-IVF and Sub-NC groups. In the two IVF groups, TTP was longer (COH-IVF4.0 years; MNC-IVF3.8 years) compared with the Sub-NC group (2.0 years). The underlying cause of parental subfertility in the IVF groups was more often paternal, whereas in the Sub-NC group, it was more often unexplained. Gestational age was shorter and birthweight was lower in the COH-IVF group than in the Sub-NC group. Singletons in the MNC-IVF group were more often small-for-gestational age compared with COH-IVF singletons. The rate of folic acid use was higher in the MNC-IVF group than in the Sub-NC group.

**Table 1:** Characteristics of participating parents and children

Characteristics	COH-IVF (*n* = 95)	MNC-IVF (*n* = 48)	Sub-NC (*n* = 68)
Parental characteristics			
Maternal age at conception, median (range)	**35.9 (27.0–41.0)** ^*/#^	**32.9 (26.2–37.5)** ^*^	**33.5 (23.1–40.3)** ^**#**^
Paternal age at conception, median (range)	**36.7 (27.5–59.3)** ^*/#^	**34.2 (28.3–47.8)** ^*^	**35.4 (25.5–48.7)** ^**#**^
Education level mother high, *n* (%)^a,b^	43 (45)	21 (44)	32 (47)
Maternal BMI before pregnancy, median (range)^a^	23.6 (17.9–42.5)	23.1 (16.8–30.6)	23.2 (18.0–46.7)
≥ 1 parent with asthma, *n* (%)	3 (3)	3 (6)	6 (9)
≥ 1 currently smoking parent, *n* (%)	22 (23)	18 (38)	18 (27)
Fertility parameters			
TTP in years, median (range)	**3.9 (0.1–13.3)** ^**#**^	**3.8 (0.1–7.5)** ^**^**^	**2.0 (0.1–11.3)** ^**#/^**^
ICSI, *n* (%)	54 (56)	23 (48)	n.a.
Maternal subfertility, *n* (%)	36 (38)	15 (31)	19 (28)
Paternal subfertility, *n* (%)	**48 (51)** ^*/#^	**26 (54)** ^*^	**18 (26)** ^**#**^
Unexplained subfertility, *n* (%)	**18 (19)** ^*/#^	**8 (17)** ^*^	**36 (58)** ^**#**^
Gestational characteristics			
Smoking during pregnancy, *n* (%)	8 (9)	5 (10)	7 (10)
Caesarean section, *n* (%)	27 (28)	8 (17)	19 (28)
Use of folic acid during pregnancy, *n* (%)	83 (91)	**48 (100)** ^**^**^	**59 (87)** ^**^**^
The use of high folic acid ≥ 5 mg, *n* (%)	10 (11)	5 (10)	2 (3)
Birth characteristics			
Gestational age in weeks, median (range)	**39.4 (33.4–42.3)** ^**#**^	39.8 (34.6–42.6)	**40.0 (30.1–42.6)** ^**#**^
Preterm birth (< 37 weeks), *n* (%)	7 (7)	6 (13)	4 (6)
Birthweight in grams, mean (*σ*)	**3385 (573)** ^**#**^	3390 (594)	**3591 (513)** ^**#**^
Low birthweight, *n* (%)	4 (4)	4 (8)	2 (3)
Small-for-gestational age, *n* (%)	**0 (0)** ^*^	**3 (6)** ^*^	1 (2)
Neonatal characteristics			
NICU admission, *n* (%)	2 (2)	2 (4)	4 (6)
Apgar score at 5 min < 7, *n* (%)^a^	1 (1)	0 (0)	0 (0)
Breastfed for > 6 weeks, *n* (%)^a^	40 (56)	22 (46)	34 (51)
Child characteristics			
Male sex, *n* (%)	51 (54)	22 (46)	34 (50)
Firstborn, *n* (%)^b^	62 (65)	34 (71)	40 (59)
Age at examination in years, median (range)	9.2 (9.0–9.7)	9.2 (9.0–10.7)	9.2 (8.4–9.9)

Statistically significant differences (*p* < 0.05) are displayed in bold. The symbols denote which groups differ significantly from each other (asterisks: ^*^; carets: ^^^; and hashtags: ^#^). Values are number (percentage), mean (standard deviation (*σ*)), or median (range)

*BMI* body mass index, *COH-IVF* controlled ovarian hyperstimulation-IVF, *ICSI* intracytoplasmic sperm injection, *MNC-IVF* modified natural cycle-IVF, *n.a.* not available, *NICU* neonatal intensive care unit, *Sub-NC* naturally conceived children born to subfertile couples, *TTP* time to pregnancy

^a^Missing data in the COH-IVF group: breastfed for > 6 weeks *n* = 23; education level father high *n* = 3; maternal BMI *n* = 5; paternal age at conception *n* = 14; smoking during pregnancy *n* = 2; the use of high folic acid ≥5 mg *n* = 4; use of folic acid during pregnancy *n* = 4. Missing data in the MNC-IVF group: Apgar score 5 min < 7 *n* = 1, paternal age at conception *n* = 1. Missing data in the Sub-NC group: Apgar score 5 min < 7 *n* = 1; breastfed for > 6 weeks *n* = 1; education level father high *n* = 1

^b^Higher vocational education or university education

### Asthma and rhinitis

Table 2 presents the asthma and rhinitis data. Asthma prevalence did not differ between the three groups. In the COH-IVF group 8 (8%), in the MNC-IVF group 0 (0%), and in the Sub-NC 4 (6%), children had asthma. The prevalence of asthma medication use also did not differ between the three groups. Adjustment for confounders did not alter the results (Table 3).

**Table 2:** Asthma and rhinitis at 9 years in the three study groups

	COH-IVF (*n* = 95)	MNC-IVF (*n* = 48)	Sub-NC (*n* = 68)
Asthma			
Asthma ever, *n* (%)	11 (12)	2 (4)	7 (10)
If so, diagnosed by physician, *n* (%)	11 (12)	2 (4)	7 (10)
Wheezing complaints last year, *n* (%)	5 (5)	0 (0)	3 (4)
Asthma medication use last year, *n* (%)	8 (8)	0 (0)	2 (3)
Current asthma, *n* (%)	8 (8)	0 (0)	4 (6)
Rhinitis			
Rhinitis ever, *n* (%)	24 (25)	9 (19)	19 (28)
Rhinitis in the past year, *n* (%)	22 (23)	7 (15)	16 (24)
From February to May, *n* (%)	4 (4)	0 (0)	1 (1)
From May to August, *n* (%)	2 (2)	1 (2)	4 (6)
All year, *n* (%)	5 (5)	0 (0)	3 (4)
No indications of months, *n* (%)	11 (12)	6 (13)	8 (12)
With itchy-watery eyes, *n* (%)	7 (7)	1 (2)	8 (12)
Interfering with daily activities, *n* (%)	6 (6)	2 (4)	2 (3)
Hay fever ever, *n* (%)	**14 (15)** ^*^	**1 (2)** ^*/#^	**10 (15)** ^**#**^
Current rhinitis, *n* (%)	7 (7)	1 (2)	8 (12)

Statistically significant differences (*p* < 0.05) are displayed in bold; the symbols denote which groups differ significantly from each other (asterisks: ^*^ and hashtags: ^#^). Values are number (percentage)

*COH-IVF* controlled ovarian hyperstimulation-IVF, *MNC-IVF* modified natural cycle-IVF, *Sub-NC* naturally conceived children born to subfertile couples

**Table 3:** Multiple regression analysis of the effect of ovarian hyperstimulation, the in vitro procedure, and a combination of these two on asthma and rhinitis

	COH-IVF vs. MNC-IVF		MNC-IVF vs. Sub-NC		COH-IVF/ICSI vs. Sub-NC	
	Adjusted OR (95% CI)	*p* value	Adjusted OR (95% CI)	*p* value	Adjusted OR (95% CI)	*p* value
Asthma						
Asthma ever	3.32 (0.65, 16.9)	0.148	0.18 (0.03, 1.21)	0.077	0.91 (0.29, 2.84)	0.911
If so, diagnosed by physician	xx	xx	xx	xx	xx	xx
Wheezing complaints last year	xx	xx	xx	xx	0.88 (0.17, 4.53)	0.877
Asthma medication use last year	xx	xx	xx	xx	2.47 (0.46, 13.4)	0.294
Current asthma	xx	xx	xx	xx	1.30(0.32, 5.24)	0.709
Rhinitis						
Rhinitis ever	1.79 (0.72, 4.45)	0.213	0.91 (0.38, 2.14)	0.823	0.84 (0.39, 1.83)	0.668
Rhinitis in the past year	4.41 (0.38, 50.7)	0.402	2.16 (0.25, 18.48)	0.484	3.22 (0.38, 27.4)	0.284
From February to May	xx	xx	xx	xx	xx	xx
From May to August	0.36 (0.53, 8.45)	0.528	0.32 (0.02, 4.22)	0.387	0.27 (0.03, 2.30)	0.427
All year	xx	xx	xx	xx	2.36 (0.34, 16.2)	0.762
No indications of months	0.17 (0.02, 1.86)	0.147	10.49 (0.57, 194.5)	0.115	0.63 (0.13, 3.02)	0.563
With itchy-watery eyes	4.02 (0.23, 70.5)	0.341	0.10 (0.01, 2.19)	0.145	0.41 (0.09, 1.95)	0.260
Interfering with daily activities	1.06 (0.08, 14.2)	0.963	3.14 (0.04, 252.2)	0.609	2.30 (0.31, 17.1)	0.416
Hay fever, ever	**9.59 (1.16, 79.0)**	**0.036**	**0.20 (0.01, 0.64)**	**0.020**	1.04 (0.39, 2.82)	0.932
Current rhinitis	4.37 (0.45, 42.2)	0.203	**0.09 (0.01, 0.90)**	**0.039**	0.48 (0.14, 1.62)	0.237

In the adjusted analyses, we corrected for a currently smoking parent, at least one parent with asthma, birthweight, maternal body mass index, and time to pregnancy. Statistically significant differences (*p* < 0.05) are displayed in bold

*COH-IVF* controlled ovarian hyperstimulation-IVF, *MNC-IVF* modified natural cycle-IVF, *Sub-NC* naturally conceived children born to subfertile couples

^a^Some confidence intervals were maximal width owing to the small numbers in the cells. Results should therefore be interpreted with caution

^b^The symbol ‘xx’ denotes that numbers were too small for regression analysis

The frequency of rhinitis ever and current rhinitis was similar in the three groups. After correction for confounders, rhinitis occurred more often in the Sub-NC group than in the MNC-IVF group. Hay fever did differ between the groups: it occurred more often in the COH-IVF group (15%) and Sub-NC group (15%) than in the MNC-IVF group (2%). This group difference in hay fever between the three groups remained after adjustment for confounders (Table 3). Additional adjustment for breastfeeding longer than 6 weeks, Caesarean section, maternal age, and the use of a high dose of folic acid (≥ 5 mg) did not alter the results.

## Discussion

This prospective follow-up study suggests that asthma and rhinitis in 9-year-old children were not associated with ovarian hyperstimulation or the in vitro culture procedure.

Our current findings at 9 years of age differ from with our follow-up data at the age of 4 years. At 4 years, we found that children born following COH-IVF more often used asthma medication than naturally conceived children of subfertile couples [8]. At that time, however, we did not use a standardized and validated questionnaire, but focused on the use of asthma medication and asthma-related symptoms. As recurrent respiratory symptoms such as cough and wheeze are highly prevalent in that age group, as they also occur during viral respiratory tract infections, asthma at age 4 is difficult to diagnose. Therefore, we interpret the results obtained in our current follow-up study as more valid.

Carson et al. used the ISAAC questionnaire to assess asthma prevalence and asthma medication use in 5- and 7-year-old IVF children and naturally conceived children of fertile and subfertile couples in the Millennium Cohort Study (*n* = 18,818) in the UK [2]. They reported an increased prevalence of asthma in 5-year-old children born after ART (adjusted OR (95% CI)—2.38 (1.34, 4.24)). At 7 years, the effect had decreased (adjusted OR (95% CI)—1.84 (1.03, 3.28)). It should be noted that in the 5-year-data, the authors adjusted for, among other things, gestational age, Caesarean section, and breastfeeding, whereas in the 7-year-data, such an adjustment was not performed. No adjustment was made for low birthweight which is a known risk factor for the development of asthma [2, 5]. In addition, Carson and colleagues found some evidence that subfertility is associated with an increased risk of asthma. Our study suggests that both IVF components, ovarian hyperstimulation, and in vitro culture procedures are not associated with asthma and asthma medication use.

This finding is in line with Källén et al., who used asthma medication as an outcome parameter recorded in a large Swedish birth register cohort aged 2 to 25 years (*n* = 2,628,728). Their data indicated that the association between IVF and asthma was mainly caused by the underlying fertility problems, rather than by IVF [7]. In addition, the study did not adjust for some risk factors known to be associated with the development of asthma such as low birthweight.

The prevalence of asthma of 0–8% (overall 6%) in our study is somewhat lower than the 11% reported in the Dutch PIAMA study (*n* = 3963) in children aged 7–8 years [3]. Also, Carson et al. reported lower prevalences than in their general population, which was attributed to the favourable background characteristics of IVF couples, such as higher educational attainment [2]. This also holds true for our study. The favourable background in our study groups is also reflected in their living conditions: according to their postal codes, they lived in areas with very low rates of air pollution [14]. Still the current prevalences of asthma, hay fever, and rhinitis are remarkably low. This underlines the notion that our study groups are not representative of the general population.

Strengths of our study are the use of the validated ISAAC-based questionnaire and the study’s design which allows to separately evaluate the effect of the in vitro culture procedure and ovarian hyperstimulation on child health. The subfertile control group (Sub-NC group) prevents overestimation of the effect of IVF.

A number of caveats need to be discussed regarding the present study. Firstly, the size of the three groups is relatively small (illustrated by the broad confidence intervals), especially the MNC-IVF group. This prevents us from drawing firm conclusions and excluding a type II error. However, other studies investigating the effect of IVF on asthma in the offspring do not have the ability to study the effect of ovarian hyperstimulation and the in vitro culture procedure separately.

Secondly, the absence of a fertile control group precludes a conclusion on the effect of the subfertility. With the couples’ TTP, we have detailed information on the duration of subfertility. TTP was included as a confounder in the regression analyses, thus making sure that our results were not confounded by the severity of subfertility. However, due to the lack of a group of children born to fertile couples, we are not able to address the impact of subfertility per se on asthma in the offspring, and therefore, we cannot rule out a potential effect of parental subfertility on the offspring’s risk for asthma.

In conclusion, our study suggests that ovarian hyperstimulation, the in vitro culture procedure, and a combination of these two in IVF with hyperstimulation are not associated with asthma and rhinitis at 9 years of age. The previously found association between COH-IVF and asthma (use of asthma medication) at 4 years of age could not be replicated at age 9. In addition, our data suggest that IVF offspring does not have a higher prevalence of asthma than children of subfertile couples conceived naturally. As ARTs are still increasingly used, it is of importance that a meta-analysis and future studies further investigate the effect of parental subfertility and the different aspects of IVF on child health, including asthma and rhinitis.

## Electronic supplementary material

ESM 1 (DOCX 61 kb)

## Abbreviations

AUMC: Amsterdam University Medical Center
COH: Controlled ovarian hyperstimulation
ISAAC: International Study of Asthma and Allergies
MNC: Modified natural cycle
PIAMA: Prevention and Incidence of Asthma and Mite Allergy
PGS: Preimplantation genetic screening
Sub-NC: Subfertile-naturally conceived
TTP: Time to pregnancy
UMCG: University Medical Center Groningen
