# Supplementary material for: Asthma in 9-year-old children of subfertile couples is not associated with in vitro fertilization procedures
Source: Eur J Pediatr. 2019 Aug 6;178(10):1493–9. doi: 10.1007/s00431-019-03436-2 (PMC6733816; doi:10.1007/s00431-019-03436-2)
Supplement: Supplementary file 1 — (DOCX 61 kb) [file 431_2019_3436_MOESM1_ESM.docx]

**Figure I: flow chart of the Groningen ART cohort study**

Children assessed at age 4 years (n=53)
♦Non participants (n=4)
 Assessment burden (n=3
 Untraceable (n=1)

Children assessed at age 4 years (n=79)
♦Non participants (n=11)
 Assessment burden (n=9)
 Moved abroad (n=1)
 Untraceable (n=1)

Children assessed at age 4 (n=105 )
♦Non participants (n=20)
 Withdrawal consent (n=1)
 Assessment burden (n=14)
 Moved abroad (n=3)
 Untraceable (n=2)

Children assessed at age 9 years (n=48)
♦Non participants (n=5)
 Assessment burden (n=3)
 Untraceable (n=2)

Children assessed at age 9 years (n=68)
♦Non participants (n=11)
 Assessment burden (n=6)
 Moved abroad (n=1)
 Untraceable (n=4)

Children assessed at age 9 years (n=95)
♦Non participants (n=10)
 Assessment burden (n=6)
 Untraceable (n=4)

Included neonates (n=125)
♦Non participants (n=21)
 Declined to participate (n=21)

Included neonates (n=90)
♦Non participants (n=53)
Declined to participate (n=53)

Included neonates (n=57)
♦Non participants (n=22)
 Declined to participate (n=22)

MNC-IVF n=79

Sub NC n=143

COH-IVF (Total) n=146

COH-IVF (Groningen ART cohort) n= 89

COH-IVF (PGS-trial) n= 57

CO

Live born infants fulfilling the inclusion criteria (n=368)
